# Sexual and reproductive health needs assessment and interventions in a female psychiatric intensive care unit

**DOI:** 10.1192/bjb.2021.107

**Published:** 2023-02

**Authors:** Elana Covshoff, Lucy Blake, Elizabeth Mary Rose, Adenike Bolade, Robert Rathouse, Aleishia Wilson, Arthur Cotterell, Rudiger Pittrof, Faisil Sethi

**Affiliations:** 1 Guy's and St Thomas’ NHS Foundation Trust, UK; 2 South London and Maudsley NHS Foundation Trust, UK; 3 Dorset HealthCare University NHS Foundation Trust, UK

**Keywords:** In-patient treatment, sexual and gender identity disorders, education and training, human rights, psychiatric nursing

## Abstract

**Aims and method:**

To assess the sexual and reproductive health (SRH) needs of women admitted to a psychiatric intensive care unit (PICU), and acceptability of delivering specialist SRH assessments and interventions in this setting. Within a quality improvement framework, staff were trained, a clinical protocol developed and clinical interventions made accessible.

**Results:**

Thirty per cent of women were identified as having unmet SRH needs and proceeded to a specialist appointment, representing a 2.5-fold increase in unmet need detection. Forty-two per cent of women were assessed, representing a 3.5-fold increase in uptake. Twenty-one per cent of women initiated SRH interventions, of which 14% had all their SRH needs met. Staff, patients and carers highlighted the acceptability and importance of SRH care, if interventions were appropriately timed and patients’ individual risk profiles were considered. Barriers to access included lack of routine enquiry, illness acuity and impact of the COVID-19 pandemic.

**Clinical implications:**

SRH needs for PICU admissions are greater than previously realised. Providing a nurse-led SRH assessment is acceptable, feasible and beneficial for PICU patients.

People with serious mental illness experience significant health inequalities compared with the general population.^1,2^ These include reduced access to sexual and reproductive health (SRH) services, resulting in unmet contraceptive needs, a higher prevalence of sexually transmitted infections (STIs) and sexual dysfunction.^3–7^ Psychiatric intensive care units (PICUs) provide specialist in-patient treatment to patients with severe mental disorders whose complex needs cannot be managed in a general psychiatric setting. Such needs include physical health comorbidities^8^ and clinical risk management. Patients in PICUs are acutely unwell from a mental and often physical health perspective, therefore their clinical state, risk profile, engagement and capacity are highly fluctuant, which can present significant challenges.

In recent years, there has been a growing drive for parity of esteem between mental and physical health. This transformative journey, acknowledged in the Independent Mental Health Taskforce's ‘Five Year Forward View for Mental Health’ report,^9^ aims to bring the mind closer to the body and reduce inequalities. Women admitted to the PICU view both their physical and mental health needs as a priority.^10^ In practice, overcoming barriers to accessing physical healthcare in a PICU setting can be challenging across interfaces, and often requires innovation.

This quality improvement project was a collaboration between an adult, in-patient female PICU in a National Health Service (NHS) Foundation Trust (see https://www.slam.nhs.uk) in South London and the Sexual and Reproductive Health Rights, Inclusion and Empowerment (SHRINE) programme.^11^ SHRINE is a London-based programme delivering SRH care to any individual with serious mental illness, substance misuse and/or intellectual disability. SRH assessments (via SHRINE) had been generally available to patients in the wider system since 2016, and to the female PICU patients since mid-2018. However, a retrospective analysis of 15 month of activity data found that only 25 SHRINE referrals had been made across 205 female PICU admissions. This low referral rate of 12% likely reflected pathway barriers and was unlikely to represent the actual clinical need in female PICU patients.

## Aim

The primary aim of this quality improvement project was to assess patients’ SRH needs, and the acceptability of providing SRH assessments in a female PICU setting. Secondary aims were to explore the barriers to access and the feasibility of providing SRH assessments and SHRINE interventions in the PICU.

## Governance and ethics

This was an NHS Trust registered quality improvement project linked to a pre-existing service offer, and formal ethical approval was not required. It is important to note that the clinical perspectives in sexual health medicine routinely lead to ethical considerations as part of standard clinical practice. Such clinical considerations were framed with reference to human rights, the World Health Organization's AAAQ (Availability, Accessibility, Acceptability, Quality) Framework^12^ and the Fairness, Respect, Equality, Dignity and Autonomy principles,^13^ to ensure dignity and autonomy were prioritised.

## Method

The SHRINE programme was a system-wide offer in place since 2016, and the PICU link to SHRINE was initially developed in 2018. Our retrospective analysis found an ineffective referral pathway leading to poor utilisation of the pre-existing clinical service offer. This quality improvement project was initiated to improve access and quality of SRH care.

Between September and November 2019, multidisciplinary staff focus groups were held and patients were consulted via PICU community groups to understand the barriers to access and feasibility. Themes included the clinical risk profile of PICU patients, the timing of SRH interventions in the PICU care pathway, the PICU team's training needs and assessment competencies for assessing high-risk sexual behaviours.

Drawing from these themes, the multidisciplinary team developed a new clinical protocol incorporating a fresh approach to referral and assessment. This included a checklist of SRH needs to guide SRH conversations. The SHRINE programme's visibility was enhanced on the PICU with the use of welcome packs and fuller integration into standard clinical assessment processes. A key change was to inquire why at times patients and staff may consider a SHRINE referral or intervention unsuitable.

**Fig. 1:**
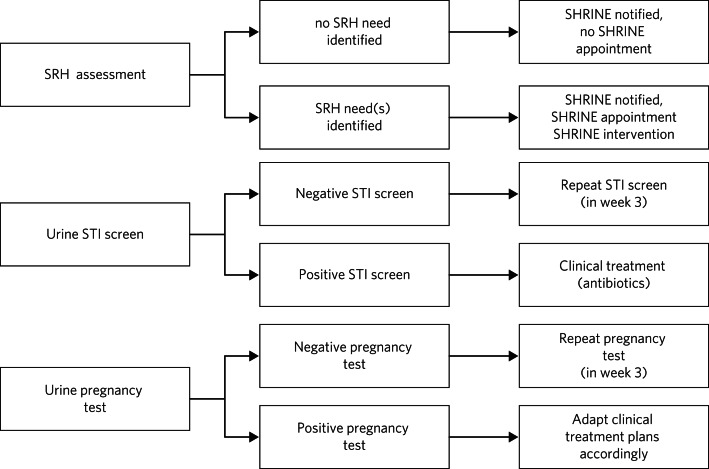
Clinical assessments. SHRINE, Sexual and Reproductive Health Rights, Inclusion and Empowerment programme; SRH, sexual and reproductive health; STI, sexually transmitted infection.

In line with the quality improvement approach, the clinical protocol was continuously improved based on feedback and learning in multiple PDSA (Plan, Do, Study, Act)^14^ cycles over the course of the project (Fig. 1). The clinical protocol required nursing staff to attempt an SRH assessment alongside an STI screen and a urine pregnancy test. These were incorporated within the routine physical health assessment on admission to the PICU. A 2-week window was considered acceptable for attempted completion of these clinical tasks, to allow for fluctuations in patients’ mental state, capacity to consent and risk profiles. One key change was the additional testing for STIs and pregnancy 3 weeks after admission, to reduce the risk of false negatives or the probability of missed pregnancy linked to pre-admission sexual activity.

Alongside clinical protocol development, staff were trained in a variety of SRH topics, including contraception choices, HIV and STIs. The effect of staff training was assessed by online pre- and post-training questionnaires.

Having implemented the new clinical protocol, data for both referrals and interventions/outcomes was monitored between December 2019 and June 2020. This data was compared with the crude baseline provided by our retrospective activity analysis. Further qualitative feedback via survey captured patients’ and carers’ experience as part of our overall evaluation.

## Results

### Clinical picture

The pre-project referral rate of 12% may be seen as a crude baseline figure for the level of SRH assessments conducted on the female PICU ward. This figure assumes that reactive *ad hoc* SRH care reflects the true level of need, which is an unreliable assumption.

Between December 2019 and June 2020, there were a total of 77 PICU admissions (female PICU sample group). Most of these admissions involved a manic crisis (*n* = 46; 59.7%) associated with a variety of diagnostic aetiologies. The remaining admissions presented with (non-manic) psychotic states (*n* = 24; 31.2%) or personality difficulties/disorder (*n* = 7; 9.1%).

Of the 77 PICU admissions within the project data timeframe, 41.6% (*n* = 32) received an SRH assessment within the new ward-based clinical protocol. If 12% is the crude baseline, then this 3.5-fold increase in SRH assessment rates is indicative of the positive effect of a proactive and systematic approach to assessment and screening. Of note, 58.4% (*n* = 45) were not able to have an SRH assessment as per the new clinical protocol; this was primarily because of patients being acutely unwell in the PICU and their relatively short length of stay. Offering SRH assessments was also an entirely new process, and it took time and consistent promotion to embed the new protocol.

Of the 32 patients receiving an SRH assessment, 23 were identified as having unmet SRH needs; this equates to 29.9% of the PICU sample group or 71.9% of patients who had an SRH assessment completed. If 12% is the crude baseline, then this is at least a 2.5-fold increase in SRH unmet need detection, and it may be as high as 6.0-fold if all PICU patients were able to have an SRH assessment.

Within the subgroup of 23 patients identified as having unmet SRH needs, 65.2% (*n* = 15) were admitted to the PICU in the context of an episode of manic crisis, 21.7% (*n* = 5) with a (non-manic) psychotic state and 13.0% (*n* = 3) with personality difficulties/disorder.

**Fig. 2:**
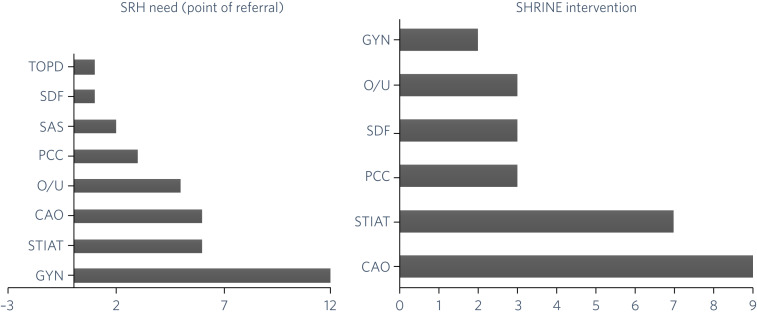
SRH needs and SHRINE interventions. CAO, contraception advice/options; GYN, gynaecology care; O/U, other (including counselling, signposting, unknown); PCC, preconception care; SAS, sexual assault support; SDF, sexual dysfunction/function; SHRINE, Sexual and Reproductive Health Rights, Inclusion and Empowerment programme; SRH, sexual and reproductive health; STIAT, sexually transmitted infection advice/testing; TOPD, termination of pregnancy discussion.

The SRH needs captured by the SRH assessment process and the associated SHRINE interventions provided in the SHRINE clinic are outlined in Figure 2. Individual patients sometimes had more than one SRH need, reflecting the vulnerability of this patient group to SRH issues; and across the subgroup of 23 patients, 36 SRH areas of need were identified at the point of referral. The most common SRH needs were gynaecological issues (such as period problems, pelvic pain and vaginal discharge), STI advice/testing and contraception advice/options.

Twenty-three patients progressed to an initial SHRINE appointment. Sixteen of these patients (20.8% of the PICU sample group) proceeded to initiate SHRINE interventions. Seven patients (9.1% of the PICU sample group) did not proceed because they lacked capacity or provided capacitous refusal. The challenges with patient capacity are unsurprising given the acuity and severity of psychiatric presentations in the PICU setting. Eleven out of the 16 patients (14.3% of the PICU sample group) completed several SHRINE interventions, most commonly in the areas of contraception advice/family planning and STI advice/testing. Some of the 11 patients who completed SHRINE interventions, did so across more than one appointment, and many received several SHRINE interventions. For example, six patients received a contraception consultation, and three of these patients (50.0%) progressed to using a long-acting reversible contraception as their method of their choice. Four patients were offered STI testing, and three of these patients (75.0%) received an STI screen.

Five patients were unable to complete the SHRINE interventions, in major part because of the global COVID-19 pandemic and the challenges this created for direct clinical care.

**Fig. 3:**
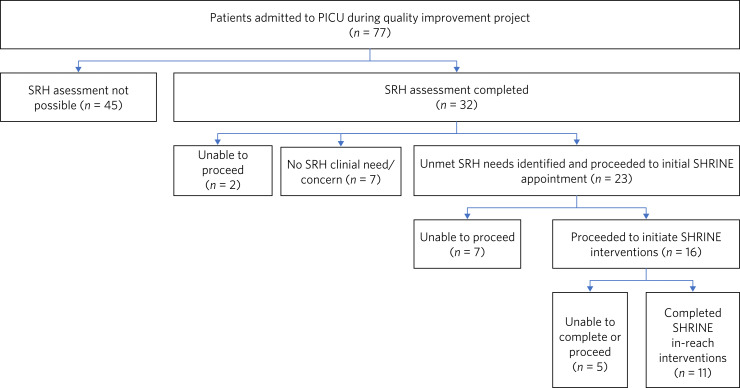
Patient flow. PICU, psychiatric intensive care unit; SRH, sexual and reproductive health; SHRINE, Sexual and Reproductive Health Rights, Inclusion and Empowerment programme.

Figure 3 displays the patient flow through the quality improvement project.

### Staff training

In the early stages of the project, 16 members of the PICU multidisciplinary team completed an online questionnaire exploring baseline knowledge and understanding. A total of 56.3% (*n* = 9) of respondents reported being confident to start the conversation about sexual health with patients, and 75.0% (*n* = 12) felt confident discussing healthy sexual relationships with patients. Discussing these topics with families and carers of patients was perceived as more challenging, with 62.5% (*n* = 10) of respondents not feeling confident. A total of 81.3% (*n* = 13) of respondents wanted training on discussing contraception and how to assess for risky behaviours. These knowledge and skills gaps were addressed through bespoke training sessions in targeted areas.

In the final stages of the project, 18 members of the PICU multidisciplinary team completed an online questionnaire (not all respondents answered all questions); 81.3% (*n* = 13) of respondents reported being confident to start the conversation about sexual health and 87.5% (*n* = 14) felt confident to discuss healthy sexual relationships with patients. This represented quite a positive shift in staff confidence post-training. Of note, at the end of the project, 75.0% (*n* = 12) of respondents felt confident discussing these topics with families and carers. The bespoke training sessions on contraception, STIs and how to assess risky behaviours were highly successful in improving staff confidence. On a scale of 0–10 (with 10 being high), after the training, 81.3% (*n* = 13) of respondents rated their confidence as 8 or above, in relation to discussing contraception/STIs; pre-training, this was 25.0% (*n* = 4). After training, 93.8% (*n* = 15) of respondents rated their confidence as 8 or above, in relation to discussing risky behaviours; pre-training, this was 18.8% (*n* = 3).

### Patient and carer feedback

Alongside other indirect mechanisms of feedback, five patients and six carers provided direct feedback via questionnaires. All 11 participants felt it was important in general to have a forum to talk about SRH, and eight (72.7%) agreed it was important in the PICU. Four of the patient participants said they might not always feel comfortable discussing SRH with PICU clinicians. The reasons given included lack of privacy in the clinical setting, preferring to speak with female clinicians and/or doctors, and being concerned about a lack of relevance to their psychiatric care. Conversely, the patient feedback highlighted a willingness to talk to an SRH specialist when in the PICU.

Specific needs identified by patient participants included discussing how their medications might affect their SRH, support with reducing impulsivity and having a general SRH check-up. Four of the patient participants had previously accessed mainstream local SRH services, with the impression given that these were one-off contacts.

Five (83.3%) of the carer participants felt it was definitely important to discuss SRH (regardless of setting). Some felt it was important to discuss when in the PICU, whereas others were unsure as they were concerned patients would be too unwell to engage or it may not be relevant in the psychiatric setting. Carer participants were concerned around the increased vulnerability of patients in relation to SRH before PICU admission, and they felt if patients were receptive and able to engage, then addressing SRH concerns would be beneficial.

## Discussion

### Barriers to accessing SRH care

People with severe mental illness experience barriers to access as well as lower-quality care, which can result in poorer SRH outcomes.^1–5^ The reasons for this may include patient factors such as vulnerability, illness-related behaviours, and comorbid substance use; staff factors such as a lack of routine enquiry of needs by mental health staff¹, knowledge and skills gaps in SRH and associated stigma; and service factors such as variable access to appropriate healthcare at times of acute illness. Given this complexity, standard service models can find it extremely challenging to prioritise and meet the SRH needs of patients; quality improvement and innovation is often required to meet these and other physical health needs for patients.

From a clinical perspective, mental illness-related behaviours can themselves result in increased SRH need (for example, because of sexual disinhibition in the context of mania), and untreated disease as well as the iatrogenic effects of psychotropic medications can affect sexual function, reproductive health and fertility. Rates of unintended and unwanted pregnancy have been found to be higher in women with severe mental illness,^15^ and those with an unintended pregnancy that goes to term may be unable to care for their child, resulting in the subsequent removal from the mother. This can lead to a devastating cycle of trauma that, in turn, may increase risk of comorbid mental health problems.^16^ Barriers to accessing SRH services where a termination of pregnancy is desired may also affect current and future well-being. Relationship, sexual and reproductive difficulties can also precipitate or perpetuate mental health difficulties.^17^ This context highlights the necessity of enhancing the profile and priority of the SRH needs in patients with severe mental illness.

### Our integrated care model

To the best of our knowledge, this is the first time that an SRH service has directly integrated into a women's PICU. The quality improvement approach, the enhancements to the skills of the PICU multidisciplinary team, and the new clinical protocol allowed for the implementation of a routine and proactive SRH assessment system, resulting in increased assessment and identification of unmet needs, as well as successful completion of interventions in a proportion of patients. However, given that 58.4% of PICU patients did not receive an SRH assessment, the true level of unmet need is likely to be much higher. Furthermore, progress to the SHRINE clinic and interventions were impeded by clinical factors, including fluctuating capacity to consent and relatively short length of stay on the ward. It is also important to note that limitations were placed on face-to-face clinical care because of the COVID-19 pandemic. This resulted in less SHRINE interventions being undertaken, likely leading to an underestimation of the level of SRH need as well as limited care. Of note, patients were not just offered support and follow-up within the framework of this project, but were also followed up post-discharge, in line with standards of good clinical care.

Before this project, clinicians were less likely to routinely explore SRH concerns with a patient unless there was a clear need, such as the disclosure of sexual assault. In these cases, conversations were often led by a doctor. The project enhanced the knowledge and consultation skills of the team in line with a new protocol, resulting in most SRH conversations being nurse-led in the early stages of the PICU admission. Nursing staff that received training felt more confident in initiating SRH conversations and were well placed to appropriately time the interventions by keeping each patient's clinical and risk status in mind.

The multidisciplinary team found that SRH assessments were best conducted several days or a couple of weeks into the PICU admission, allowing time for clinical improvement and engagement. An individualised approach was required, particularly where there were historical sexual health factors such as a history of sexual trauma, sexual disinhibition or delusions relating to SRH. In these circumstances, it was important to support and safeguard both patients and staff. All assessments and interventions were delivered with safeguarding and gender sensitivity uppermost in mind.

The positive outcomes provide reassurance that delivering nurse-led assessments is an acceptable model in this clinical environment. There were other indirect quality gains, such as that SRH assessments led to patients receiving a much wider review of health and social care needs, including endocrinology, menopause, incontinence, substance misuse, domestic violence and child health.

### Capacity challenges and delivering personalised care

The mental health in-patient setting, and particularly the PICU, may not be the obvious clinical setting to provide SRH services, but on closer inspection, it provides a unique opportunity to support patients in accessing services where they might ordinarily feel stigmatised and marginalised.^18^ Feedback from the patient and carer surveys showed that offering SRH assessments was considered both acceptable and necessary in the PICU. This feedback also highlighted that patients were responsive to discussing SRH concerns with a member of clinical staff with whom they had a good rapport and trusting relationship. In a psychiatric setting, this is hardly surprising, given the known association between therapeutic alliance and good outcomes; however, it is notable because it is in the early stages of this care journey.

In any psychiatric setting, patient capacity may be lacking or fluctuant, and clinicians will consider the necessity of immediate treatment and whether it is in the patient's best interests. There was a constant focus on patients’ capacity to consent to SRH assessments and interventions throughout this project. General considerations when weighing up the appropriateness of interventions included the clinical need and urgency, treatability of the condition, invasiveness and reversibility of the procedure, a patient's capacity to consent and whether this is likely to change. Fundamental human rights (enshrined in the Human Rights Act 1998) should be prioritised, including right to private and family life (Article 8), right to life (Article 2), right to marry and start a family (Article 12) and protection from discrimination (Article 14).^19^ Relevant ethical principles in the context of good psychiatric care include proportionality, equity, individual liberty and autonomy.

Many psychiatric in-patients are likely to regain their capacity to consent to assessments or interventions within a small number of days or weeks, with treatment of their mental disorder. In the treatment of sexually transmitted diseases, it is likely to be clinically appropriate to wait until the patient's restoration in capacity. However, if the infection is not identified through routine screening, then the potential implications on the patient's fertility cannot be addressed, even later down the line. Considerations around capacity to consent and what is in the best interests of the patient should be individualised, intervention-specific and regularly reviewed.

Given the severity of mental illness presentations in the PICU setting, there would be an understandable concern that patients would be too unwell to engage and provide capacitous consent. However, 41.6% of the PICU admissions received an SRH assessment, of which 71.9% had unmet SRH needs; 69.6% of those initiated SHRINE interventions, of which 68.8% completed SHRINE interventions. These are large percentages indicative of considerable engagement.

The multidisciplinary team's view was that a psychiatric admission should be considered an opportunity to address a patient's mental and physical health, since they are inextricably linked. Over time, with repeated and proactive engagement, patients may learn to reframe their self-worth, mitigate risky behaviours and improve their expectations of healthcare. Patients may come to understand that healthcare, including SRH, is a right to which they, like all others, are entitled.

With these factors in mind, the authors advocate for SRH needs assessments to be offered in all mental health settings, with the emphasis on identifying potential needs that can be addressed as capacity recovers.

In conclusion, there is a paucity of research both in identifying the level of SRH need in patients with severe mental illness and in assessing their ability to engage with standard services. Routine and proactive nurse-led SRH assessments were successfully implemented in this PICU setting. In doing so, the prevalence of need was far higher than expected. This finding is likely to extend to the wider psychiatric in-patient population. In psychiatric in-patient settings and especially PICUs, issues such as capacity, risk and trauma are heightened, which inevitably raises the question of the appropriateness or necessity of delivering SRH care in this context.

Data gathered on staff, patient and carer attitudes identified that all groups deemed delivery of SRH care in this PICU setting to be acceptable and even a necessary part of holistic care. Data from questionnaires and focus groups highlighted the value of training, so that staff feel sufficiently resourced and empowered to lead an intervention, ensuring these are appropriately timed.

At the very least, the implementation of routine SRH assessment in a psychiatric setting provides an opportunity for staff to engage and educate patients. We have shown it can be done, and if it is possible in a PICU, then it should be replicable in other mental health settings. At most, it may result in life changing interventions, such as the timely treatment of infection, the initiation of contraception, family planning, identification of abuse or education on maintaining good SRH and healthy relationships.

Although stark health inequalities in those with mental disorders remain, there is hope that with dynamic, innovative and individualised patient care, the barriers and stigma that prevent access to high-quality treatment can be eroded, and greater parity achieved.

## Data Availability

The authors confirm that the data supporting the findings of this study are available within the article. Further information about the quality improvement project is available on request from the corresponding author, E.C.

## About the authors

**Elana Covshoff** (MA) is the SHRINE Programme Manager at Guy's and St Thomas’ NHS Foundation Trust, UK. **Lucy Blake** (MBBS, MRCPsych) is a specialist registrar in general adult and older adult psychiatry with South London and Maudsley NHS Foundation Trust, UK. **Elizabeth Mary Rose** (MAHons, MBBS, MRCPsych) ST6 General Adult Psychiatry trainee at South London and Maudsley NHS Foundation Trust, UK. **Adenike Bolade** (Advanced Diploma in Mental Health Nursing) is the Ward Manager on ES1 Female Psychiatric Intensive Care Unit, South London and Maudsley Hospital NHS Foundation Trust, UK. **Robert Rathouse (**PGDip) is a clinical specialist occupational therapist with South London and Maudsley NHS Foundation Trust, UK. **Aleishia Wilson** (BSc) is a clinical charge nurse with South London and Maudsley NHS Foundation Trust, UK. **Arthur Cotterell** (BA (Hons), BM, BS, MRCPsych) is a Core Psychiatry trainee at South London and Maudsley NHS Foundation Trust, UK. **Rudiger Pittrof** (MSc, MRCOG, MFSRH, Dip GUM, DTM&H, FHEA) is a consultant in sexual and reproductive health with Guy's and St Thomas’ NHS Foundation Trust, UK. **Faisil Sethi** (MA (Cantab), MBBS, MScDIC, LLM, DipStat, FRCPsych) is Executive Medical Director at Dorset HealthCare University NHS Foundation Trust, UK.
